# Neonatal birth trauma: identifying new risk factors and short-term outcomes

**DOI:** 10.3389/fped.2025.1648252

**Published:** 2025-10-07

**Authors:** Nirzar Samir Parikh, Collins Odhiambo, Holleigh McMasters, Grace Kathryn Borkowski, Adam Cross, Gretchen Kopec

**Affiliations:** ^1^ Division of Neonatology, Department of Pediatrics, University of Illinois College of Medicine Peoria, Peoria, Illinois; ^2^ OSF Children's Hospital of Illinois, Peoria, Illinois; ^3^ Department of Pediatrics, University of Illinois College of Medicine Peoria, Peoria, Illinois; ^4^ University of Illinois College of Medicine Peoria, Peoria, Illinois

**Keywords:** neonates, birth trauma, social determinants of health, risk factors, outcomes

## Abstract

**Background:**

Advancements in prenatal diagnosis and obstetric care have changed the epidemiology of neonatal birth trauma in developed countries. Improving women's access to health care is key to preventing, detecting, and treating conditions that increase pregnancy complications and adverse neonatal outcomes.

**Objective:**

To identify new risk factors—focusing on social determinants of health—and short-term outcomes associated with neonatal birth trauma.

**Study design:**

Term neonates with unexpected complications born between January 1, 2019, and March 31, 2023, at 10 diverse hospitals in our health system were identified using Perinatal Care-06 coding. Maternal and neonatal charts were reviewed and recorded in REDCap. Neonates with and without birth trauma were assigned to case and control groups, respectively. Risk factors were identified using Pearson chi-square tests and multivariable logistic regression.

**Results:**

Of 711 neonates, 187 (26.3%) experienced birth trauma, primarily scalp injuries (Caput Succedaneum 42%, Ecchymosis/Bruising 27%). There were no significant differences in race, language barriers, insurance type, marital status, prenatal care access, mean household income (zip code), gestational age, maternal height, birth weight, or head circumference (all *p* > 0.05). Significant differences were observed in maternal age (*p* = 0.042), gravidity (*p* = 0.04), and parity (*p* = 0.002), with affected mothers being younger, with fewer pregnancies and lower parity. Mothers with chronic or gestational hypertension, with or without preeclampsia, had higher odds of neonatal birth trauma (OR = 1.582, 95% CI: 1.081–2.316, *p* = 0.018). Emergent deliveries nearly tripled the odds (OR = 2.8, 95% CI: 1.934–4.054, *p* < 0.001). Neonates exposed to maternal epidural anesthesia were more likely to suffer from birth trauma (77.5 vs. 51.7%, *p* < 0.001).

**Conclusion:**

Social determinants and prenatal care access did not significantly impact birth trauma. However, hypertension, exposure to epidural anesthesia and emergent delivery were associated with an increased risk.

## Introduction

Birth trauma, often interchanged with birth injury, is defined as any trauma occurring during the process of labor, delivery or neonatal resuscitation leading to structural and/or functional damage of the neonate's body (1–4). These events range from minor injuries such as skin lacerations to major life-threatening injuries such as subgaleal hemorrhage, leading to significant morbidity and mortality (2). Up to 37 birth traumas per 1,000 births have been reported in a few population-based studies conducted (4). The quality of obstetric care is often measured by the rate of birth trauma. Parents frequently believe that birth traumas can be avoided, and when they occur, it can result in feelings of anger, frustration, and, in some cases, legal action (5).

Risk factors identified for neonatal birth traumas can be categorized into maternal factors, fetal factors and the application of instrumentation during delivery (6). Maternal factors that may contribute to neonatal birth trauma include young and old maternal age, parity, poor maternal health, and abnormalities in the shape or size of pelvis. Fetal factors that may contribute to birth trauma include macrosomia, fetal weight and height, prematurity and postdates. Labor and delivery related factors include prolonged labor, fetal malpresentation and malposition, cesarean, and instrumental deliveries (7).

The epidemiology of risk factors has changed over the last two decades as the number of instrumental deliveries has decreased (8). Cesarean deliveries, a protective factor for birth trauma (9), have increased owing to increasing incidence of large for gestational age (LGA) neonates secondary to maternal obesity and gestational diabetes (10). Along with obesity, diabetes, and hypertension before and during pregnancy, the rise in cesarean deliveries has also been associated with increasing maternal morbidity (11). Cesarean deliveries have been associated with increased neonatal intensive care unit admissions (12) along with lower rates and early cessation of breast feeding secondary to delayed milk production (13) and postoperative pain (14).

Improving a woman's access to health care during her reproductive years is essential for the prevention, early detection, and treatment of conditions that could otherwise lead to pregnancy-related complications and increased infant mortality (15). Maternal morbidity rates remain disproportionately higher among Black and Hispanic women compared to their Caucasian counterparts. Similarly, women who are uninsured or enrolled in Medicaid exhibit higher morbidity rates than those with private insurance coverage (16). These disparities underscore the significant impact of social determinants of health (SDOH)—factors that influence how individuals grow, live, work, and perceive control over their environment (17). Addressing these determinants is crucial in reducing health inequities and improving maternal and neonatal outcomes across diverse populations.

Advancements in prenatal diagnosis and improvements in obstetric care have made it possible to identify risk factors for most birth traumas in neonates. However, it becomes difficult to predict and prevent birth traumas without identifiable risk factors (5). Therefore, knowledge of the pattern of birth traumas and the related risk factors can benefit both obstetricians and the pediatrician/neonatologist in case management and prognostication. Our study aims to identify new risk factors focusing on SDOH and outcomes associated with neonatal birth trauma.

## Materials and methods

### Study design and period

This retrospective cohort study investigated term neonates born at ≥37 + 0/7 weeks gestational age with unexpected complications at birth between January 1, 2019, and March 31, 2023. Data was collected from 10 diverse OSF hospitals across the states of Illinois and Michigan. Nine of the hospitals were in the state of Illinois that included St. Francis Medical Center in Peoria, St. Joseph Medical Center in Bloomington, Sacred Heart Medical Center in Danville, St. Mary Medical Center in Galesburg, Heart of Mary Medical Center in Urbana, St. James Medical Center in Pontiac, St. Elizabeth Medical Center in Ottawa, St. Anthony Medical Center in Rockford, Little Company of Mary in Chicago. The final hospital was St. Francis Hospital in Escanaba, Michigan. Maternal and neonatal medical records were thoroughly reviewed to gather comprehensive demographic, clinical, and outcome data.

### Data management

Data was securely captured in REDCap, a web-based platform managed by the University of Illinois at Chicago's Center for Clinical and Translational Science. After abstraction for 711 patients, the dataset was downloaded into Excel and subsequently exported to IBM SPSS for data cleaning and analysis. The study included all neonatal births at the participating hospitals during the study period, and no data points were excluded.

### Inclusion criteria

Term neonates born at ≥37 + 0/7 weeks gestational age at the 10 OSF hospitals. These hospitals ranged from having a Level 1 Newborn Nursery to a Level 4 neonatal intensive care unit.

### Exclusion criteria

Preterm neonates born ≤36 + 6/7 weeks gestational age. Also, those born at home or with pre-existing conditions or congenital malformations were excluded.

### Source and study population

#### Source population

Term neonates with no preexisting conditions who experienced unexpected complications were identified using the Perinatal Care-06 coding.

#### Study population

Term neonates with no preexisting conditions who experienced birth trauma.

### Outcome Variable

The primary outcome of the study was birth trauma, defined as the presence of one or more injuries sustained during delivery. These injuries were categorized (1) as follows:

#### Scalp injuries

Included cephalohematoma, caput succedaneum, subgaleal hemorrhage, and other scalp lesions.

#### Soft tissue injuries

Included erythema, abrasions, petechiae, ecchymoses (bruising), subcutaneous fat necrosis and lacerations.

#### Cerebral injuries

Included subdural, subarachnoid, tentorial, intraventricular, and intracranial hemorrhages.

#### Fractures

Included skull, clavicle and long bone fractures.

#### Spinal cord injuries

#### Brachial plexus injuries

caused by stretching of the cervical nerve roots from traction on the neck during delivery. These include upper arm palsy (Erb-Duchenne), lower arm palsy (Klumpke) and Horner's syndrome.

#### Cranial nerve injuries

Other neonatal outcomes of interest included neonatal death, need for respiratory support including continuous positive airway pressure (CPAP) or non-invasive mechanical ventilation (NIMV) or invasive mechanical ventilation, seizures, hypoxic-ischemic encephalopathy (HIE), meconium aspiration syndrome (MAS), culture-proven sepsis/meningitis, hyperbilirubinemia requiring phototherapy, pneumothorax, total length of stay, and transfer to higher-level care.

### Data collection

This retrospective study utilized patient medical records to obtain detailed maternal, labor and delivery, and neonatal variables. All data collection procedures adhered to Institutional Review Board (IRB) protocols. Data were entered into REDCap, a secure, web-based platform administered by the University of Illinois College of Medicine, and underwent rigorous cleaning to ensure completeness, accuracy, and standardized coding before statistical analysis.

**Figure 1:**
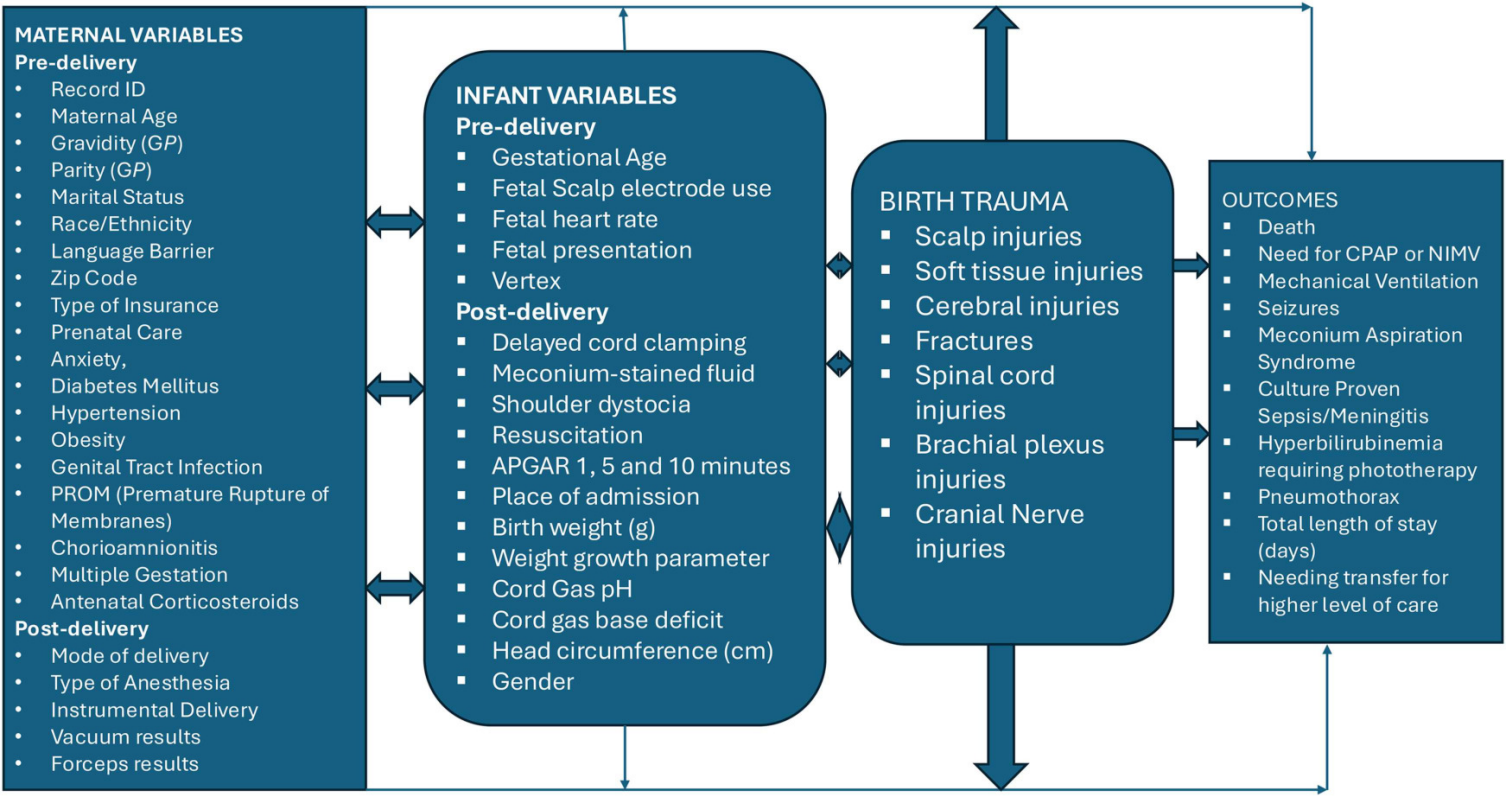
Variables and outcomes associated with birth trauma.

See details on variables in Figure 1 below.

### Data analysis

Descriptive statistics were used to summarize study variables. For continuous variables, means and standard deviations were calculated, while counts and percentages were used for categorical data. Categorical data analysis included Pearson's chi-square test assessed associations between neonatal birth trauma and categorical predictors and independent unpaired *t*-tests (with unequal variance) compared continuous variables. A multivariable logistic regression was performed using a conditional forward approach to identify predictors of birth trauma. Key confounders, including maternal age, gestational age, and prenatal care status, were adjusted in the final models. A contingency table (2 × 2) was constructed to compare the exposure and outcome variables of interest. The odds ratio (OR) and corresponding 95% confidence interval (CI) were calculated to quantify the strength and direction of the association between exposure and outcome. Given the small cell counts in the table, the exact method was applied to obtain both the OR and its CI, ensuring accurate estimation without reliance on large-sample assumptions. Fisher's exact test was used to assess statistical significance. A *p*-value <0.05 was considered statistically significant. All statistical analyses were conducted using IBM SPSS Statistics version 20.

## Results

Of the 711 records, we found 187 (26.3%) records with birth trauma. Neonates with and without birth trauma were assigned to the case and control group respectively. There were no significant differences between the groups in terms of race, language barrier, type of insurance, marital status, prenatal care, maternal height, birth weight and head circumference (all *p*-values >0.05). The independent median test was applied to compare the central tendency of gravidity and parity between participants with and without a history of trauma. This nonparametric test was chosen because these variables were expressed as medians with interquartile ranges (IQR), indicating non-normal distributions. Gravidity did not differ significantly between groups [median (IQR): 2 (2) in both; *p* = 0.064]. In contrast, parity showed a statistically significant difference, with lower median parity among those who had experienced trauma [median (IQR): 0 (2)] compared to those without trauma [median (IQR): 1 (2); *p* = 0.028] (Table 1).

### Maternal comorbidities and labor/delivery variables

There were no significant differences between the groups in terms of mothers with anxiety or depression, diabetes, obesity, genital tract infection, premature rupture of membranes, chorioamnionitis, multiple gestation, needing antenatal corticosteroids or neonatology consult (all *p*-values >0.05). However, chronic or gestational hypertension with or without pre-eclampsia was more prevalent in mothers that delivered neonates with birth trauma compared to those without birth trauma (28.9% vs. 20.4%, *p* = 0.018). The odds for mothers to be hypertensive were 1.582 times higher (95% CI: 1.081–2.316), indicating a potential association between hypertension and neonatal birth trauma (Table 2).

**Table 1:** Socio-demographic characteristics of mothers crosstabulation.

Characteristics		Trauma experience		Total	*p*-value*
		No trauma experienced	Experience any form of trauma		
Marital status, *N* (%)	Single	237 (45.4)	95 (50.8)	332 (46.8)	0.200*
	Married	267 (51.2)	86 (46.0)	353 (49.8)	
	Divorced	18 (3.4)	5 (2.7)	23 (3.2)	
	Widowed	0 (0)	1 (0.5)	1 (0.2)	
Race, *N* (%)	Caucasian	380 (72.5)	129 (69.0)	509 (71.6)	0.298*
	African American	78 (14.9)	27 (14.4)	105 (14.8)	
	Hispanic	53 (10.1)	21 (11.2)	74 (10.4)	
	Asian	10 (1.9)	6 (3.2)	16 (2.3)	
	Others	3 (0.6)	4 (2.1)	7 (1)	
Language barrier (English not the primary language), *N* (%)	No	502 (95.8)	179 (96.2)	681 (95.9)	0.797*
	Yes	22 (4.2)	7 (3.8)	29 (4.1)	
Type of Insurance, *N* (%)	Private	277 (52.9)	94 (50.3)	371 (52.2)	0.463
	Medicaid	244 (46.6)	93 (49.7)	337 (47.4)	
	Self-Pay	3 (0.6)	0 (0)	3 (0.4)	
Prenatal care, *N* (%)	Sufficient	465 (88.7)	169 (90.4)	634 (89.2)	0.701*
	Insufficient	53 (10.1)	17 (9.1)	70 (9.8)	
	None	6 (1.2)	1 (0.5)	7 (1.0)	
Gestational age, mean (SD)		39.1 (1.2)	39.2 (1.1)	39.1 (1.2)	0.388**
Maternal age, mean (SD)		28.9 (5.7)	27.8 (5.9)	28.6 (5.8)	0.042**
Gravidity, median (IQR)		2 (2)	2 (2)	2 (2)	0.064***
Parity, median (IQR)		1 (2)	0 (2)	1 (2)	<0.028***
Maternal height (cm), mean (SD)		164.2 (7.1)	163.2 (6.9)	164.0 (7.0)	0.090**
Birth weight (gm)		3498 (501)	3554.6 (500)	3512.8 (501)	0.093**
Head circumference (cm)		34.8 (1.5)	34.8 (1.5)	34.8 (1.5)	0.328**

*P*-value computation was varied i.e.

* Used either Pearson Chi-square or Fisher's Exact Test (generalized) depending on number of counts in the cells.

** Independent *t*-test.

*** Independent samples median test.

**Table 2:** Maternal comorbidities crosstabulation.

Maternal comorbidities		Trauma experience, *N* (%)		Total	*p*-value*	Odds ratio	Lower 95% CI for OR	Upper 95% CI for OR
		No trauma experienced	Experience any form of trauma					
Anxiety/Depression	No	355 (67.9)	139 (74.3)	494 (69.6)	0.100	0.730	0.501	1.063
	Yes	168 (32.1)	48 (25.7)	216 (30.4)				
Diabetes mellitus	No	448 (85.5)	153 (81.8)	601 (84.5)	0.232	1.310	0.840	2.042
	Yes	76 (14.5)	34 (18.2)	110 (15.5)				
Hypertension	No	417 (79.6)	133 (71.1)	550 (77.4)	0.018	1.582	1.081	2.316
	Yes	107 (20.4)	54 (28.9)	161 (22.6)				
Obesity	No	302 (57.7)	107 (57.2)	409 (57.6)	0.901	1.022	0.729	1.432
	Yes	221 (42.3)	80 (42.8)	301 (42.4)				
Genital tract infection	No	498 (95.4)	182 (97.3)	680 (95.9)	0.254	0.570	0.214	1.516
	Yes	24 (4.6)	5 (2.7)	29 (4.1)				
Premature rupture of membranes	No	493 (94.3)	175 (93.6)	668 (94.1)	0.735	1.127	0.564	2.250
	Yes	30 (5.7)	12 (6.4)	42 (5.9)				
Chorioamnionitis	No	490 (93.7)	177 (94.7)	667 (93.9)	0.636	0.839	0.405	1.737
	Yes	33 (6.3)	10 (5.3)	43 (6.1)				
Multiple gestation	No	524 (100)	185 (99.5)	709 (99.9)	0.093	0.261	0.231	0.295
	Yes	0 (0)	1 (0.5)	1 (0.1)				
NICU consult	No	520 (99.4)	186 (99.5)	706 (99.4)	0.951	0.932	0.096	9.014
	Yes	3 (0.6)	1 (0.5)	4 (0.6)				

*Used either Pearson Chi-square or Fisher's Exact Test (generalized) depending on number of counts in the cells.

Mothers with history of FSE electrode use had significantly higher odds of having neonates with birth trauma (OR = 2.342, 95% CI: 1.565–3.506, *p* < 0.001). There was a statistically significant association between the type of anesthesia used during delivery and the experience of birth trauma (*p* < 0.001). Notably, 77.5% of neonates who experienced any form of trauma were born to mothers who received epidural anesthesia, compared to 51.7% in the non-trauma group. None of the mothers who received general anesthesia had neonates with birth trauma, despite representing 5.2% of the non-trauma group. Smaller proportions of trauma cases were also observed among those who received spinal (10.2%), local (4.3%), or no anesthesia (8%). Neonates born via operative vaginal delivery (vacuum or forceps-assisted delivery) had significantly higher odds of having birth trauma (OR = 7.674, 95% CI: 0.951–61.949, *p* = 0.028). Emergent deliveries nearly tripled the odds of having neonates with birth trauma (OR = 2.8, 95% CI: 1.934–4.054, *p* < 0.001). Neonates with shoulder dystocia had significantly higher odds of having birth trauma (OR = 10.297, 95% CI: 5.355–19.801, *p* < 0.001). There were no significant differences between the groups in terms of fetal presentation, meconium-stained amniotic fluid and delayed cord clamping (*p*-values >0.05). Given the distribution of ACS use across trauma groups—None: 94.3%, 1 dose: 0.6%, 2+ doses: 5.2% in the no-trauma group vs. None: 93.6%, 1 dose: 0.5%, 2+ doses: 5.9% in the trauma group—and a non-significant *p*-value of 0.929, ACS use does not appear to be associated with neonatal trauma in this dataset. Among the 711 neonates, resuscitation patterns differed slightly between those who experienced birth trauma and those who did not. The most common intervention was CPAP plus drying and stimulation, used in 70% of all cases (71% no trauma vs. 67% trauma). Basic resuscitation with just drying and stimulation was more common in the trauma group (56%) than in the non-trauma group (51%). More intensive interventions, such as intubation and chest compressions, were slightly more frequent among those with trauma (e.g., 30% vs. 27% for intubation; 3% vs. 3% for chest compressions). Although higher levels of resuscitation were used in both groups, there was no major difference, suggesting a modest association between trauma experience and the intensity of neonatal resuscitation (Table 3).

**Table 3:** Labor and delivery variables crosstabulations.

Labor and delivery		Trauma experience, *N* (%)		Total	*p*-value	Odds ratio	Lower 95% CI for OR	Upper 95% CI for OR
		No trauma experienced	Experience any form of trauma					
Antenatal corticosteroids	None	494 (94.3)	175 (93.6)	669 (94.1)	0.929	–	–	–
	1 dose	3 (0.6)	1 (0.5)	4 (0.6)				
	2 or more doses	27 (5.2)	11 (5.9)	38 (5.3)				
Fetal scalp electrode use	No	450 (85.9)	135 (72.2)	585 (82.3)	0.000	2.342	1.565	3.506
	Yes	74 (14.1)	52 (27.8)	126 (17.7)				
Mode of delivery	Non-emergency	264 (78.8)	98 (88.2)	362 (81.2)	0.017	0.477	0.236	0.897
	Urgent/Emergent CS	71 (21.2)	13 (11.2)	84 (18.8)				
Type of anesthesia	General	27 (5.2)	0 (0)	27 (3.8)	<0.01	–	–	–
	Spinal	163 (31.1)	19 (10.2)	182 (25.6)				
	Epidural	271 (51.7)	145 (77.5)	416 (58.5)				
	Local	18 (3.4)	8 (4.3)	26 (3.7)				
	None	45 (8.6)	15 (8)	60 (8.4)				
Instrumental delivery	Vacuum	22 (95.7)	43 (74.1)	65 (80.2)	0.028	7.674	0.951	61.949
	Forceps	1 (4.3)	15 (25.9)	16 (19.8)				
Vacuum results	Successful	17 (77.3)	38 (88.4)	55 (84.6)	0.241	0.447	0.114	1.752
	Failed	5 (22.7)	5 (11.6)	10 (15.4)				
Inborn	No	5 (1)	1 (0.5)	6 (0.8)	0.590	1.792	0.208	15.438
	Yes	519 (99)	186 (99.5)	705 (99.2)				
Fetal presentation vertex	No	27 (5.2)	9 (4.8)	36 (5.1)	0.856	1.074	0.496	2.329
	Yes	497 (94.8)	178 (95.2)	675 (94.9)				
Delayed cord clamping	Less than 60 s	159 (30.5)	75 (40.3)	234 (33.1)	0.1041	0.747	0.526	1.059
	60 s	285 (54.6)	88 (47.3)	373 (52.7)				
Meconium-stained amniotic fluid	No	370 (71)	143 (76.9)	513 (72.6)	0.124	0.737	0.499	1.088
	Yes	151 (29)	43 (23.1)	194 (27.4)				
Shoulder dystocia	No	508 (97.5)	148 (79.1)	656 (92.7)	0.000	10.297	5.355	19.801
	Yes	13 (2.5)	39 (20.9)	52 (7.3)				

### Neonatal outcomes

**Figure 2:**
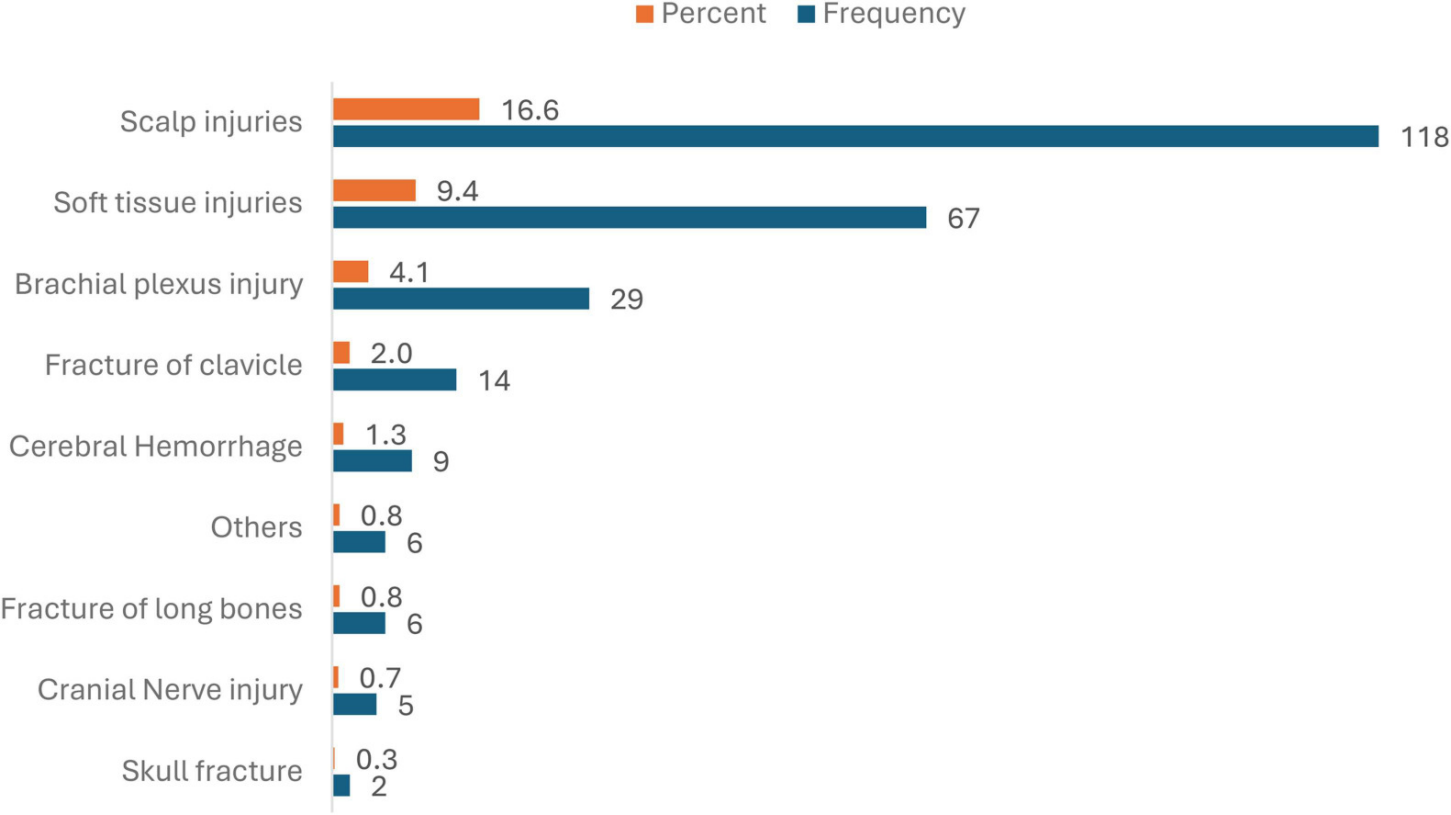
Types of birth trauma. **There were 256 birth injuries noted with 187 neonates having at least 1 birth injury. See Supplementary Table S1.

Of the 711 neonates, 187 (26.3%) experienced birth trauma, primarily scalp injuries (Caput Succedaneum 42%, Ecchymosis and Bruising 27%). These were primarily scalp injuries followed by soft tissue injuries, brachial plexus injury, clavicular fracture, others, cerebral injuries, fracture of long bones, cranial nerve injury, and skull fracture (Figure 2). There were no significant differences between the groups in terms of death, mechanical ventilation, hyperbilirubinemia needing phototherapy, seizures and HIE. Neonates with birth trauma were less likely to require respiratory support in the form of continuous positive airway pressure or non-invasive mechanical ventilation (29.9% vs. 50.1%, *p* < 0.001). Meconium Aspiration Syndrome was significantly less common among neonates who experienced any form of trauma compared to those who did not (5.9% vs. 11.8%, *p* = 0.021). The odds of experiencing trauma were reduced by more than half in the presence of MAS, with an odds ratio of 0.466. Neonates who experienced trauma were significantly less likely to develop pneumothorax compared with those without trauma (5.4% vs. 13.3%, *p* = 0.003). The odds of pneumothorax were 63% lower in this group (OR = 0.37). In contrast, neonates with birth trauma were over four times more likely to develop culture-proven sepsis or meningitis (4.3% vs. 1.0%, *p* = 0.004). We acknowledge that the higher-than-expected pneumothorax rate in our cohort may reflect contributing factors such as ventilation practices, underlying lung pathology, or selection bias. The observed association between lower pneumothorax rates and trauma cases may also be related to the involvement of more experienced providers, though this remains speculative. Those with birth trauma were less likely to require transfer to a higher-level of care facility (23% vs. 39.3%, *p* < 0.001) (Table 4).

**Table 4:** Neonatal outcomes.

Outcomes		Trauma experience		Total	*p*-value	ODDS Ratio	95% confidence interval	
		No trauma experienced	Experience any form of Trauma				Lower	Upper
Death	No	518 (99)	184 (98.4)	702 (98.9)	0.471	1.689	0.400	7.138
	Yes	5 (1)	3 (1.6)	8 (1.1)				
Need for CPAP or NIMV	No	261 (49.9)	131 (70.1)	392 (55.2)	0.000	0.426	0.298	0.608
	Yes	262 (50.1)	56 (29.9)	318 (44.8)				
Mechanical ventilation	No	487 (93.1)	176 (94.1)	663 (93.4)	0.637	0.845	0.421	1.697
	Yes	36 (6.9)	11 (5.9)	47 (6.6)				
Seizures	No	512 (97.9)	180 (96.3)	692 (97.5)	0.221	1.810	0.691	4.740
	Yes	11 (2.1)	7 (3.7)	18 (2.5)				
Hypoxic ischemic encephalopathy	No	495 (94.8)	174 (93)	669 (94.4)	0.365	1.370	0.691	2.714
	Yes	27 (5.2)	13 (7)	40 (5.6)				
Meconium Aspiration Syndrome	No	462 (88.2)	176 (94.1)	638 (89.7)	0.021	0.466	0.240	0.905
	Yes	62 (11.8)	11 (5.9)	73 (10.3)				
Culture proven sepsis/Meningitis	No	518 (99)	179 (95.7)	697 (98.2)	0.004	4.630	1.495	14.336
	Yes	5 (1)	8 (4.3)	13 (1.8)				
Hyperbilirubinemia requiring phototherapy	No	406 (77.6)	136 (72.7)	542 (76.3)	0.176	1.301	0.888	1.907
	Yes	117 (22.4)	51 (27.3)	168 (23.7)				
Pneumothorax	No	451 (86.7)	176 (94.6)	627 (88.8)	0.003	0.371	0.187	0.737
	Yes	69 (13.3)	10 (5.4)	79 (11.2)				
Needing transfer for higher level of care	No	318 (60.7)	144 (77)	462 (65)	0.000	0.461	0.314	0.676
	Yes	206 (39.3)	43 (23)	249 (35)				

**Table 5:** Logistic regression- variables in the equation.

Socio-demographic factors and maternal co-morbidities	B	S.E.	Wald	df	Sig.	Exp(B)	95% C.I. for EXP(B)	
							Lower	Upper
Marital status			1.966	3	0.580			
Marital status (Married)	−0.29	0.21	1.89	1.00	0.17	0.75	0.49	1.13
Marital status (Divorced)	−0.25	0.54	0.21	1.00	0.64	0.78	0.27	2.25
Marital status (Widowed)	21.96	–	–	–	–	–	–	
Race/Ethnicity			5.25	4.00	0.26			
Race/Ethnicity (African- American)	−0.12	0.28	0.20	1.00	0.65	0.88	0.51	1.52
Race/Ethnicity (Hispanic)	0.05	0.30	0.03	1.00	0.86	1.05	0.58	1.91
Race/Ethnicity (Asia)	0.49	0.57	0.74	1.00	0.39	1.64	0.53	5.05
Race/Ethnicity (Others)	1.67	0.81	4.24	1.00	0.04	5.33	1.08	26.27
Language Barrier (English not primary language) (1)	−0.32	0.50	0.43	1.00	0.51	0.72	0.27	1.91
Type of Insurance			0.35	2.00	0.84			
Type of insurance (1)	0.13	0.22	0.35	1.00	0.55	1.14	0.74	1.74
Type of insurance (2)	−19.98	23,203.56	0.00	1.00	1.00			
Prenatal care			0.65	2.00	0.72			
Prenatal care (1)	−0.13	0.32	0.18	1.00	0.67	0.88	0.47	1.63
Prenatal care (2)	−0.77	1.10	0.50	1.00	0.48	0.46	0.05	3.96
Anxiety/Depression (1)	−0.45	0.21	4.59	1.00	0.03	0.64	0.43	0.96
Diabetes mellitus (1)	0.20	0.25	0.68	1.00	0.41	1.23	0.76	1.99
Hypertension (1)	0.53	0.21	6.35	1.00	0.01	1.70	1.12	2.56
Obesity (1)	−0.06	0.19	0.10	1.00	0.75	0.94	0.65	1.37
Genital tract infection (1)	−0.71	0.52	1.87	1.00	0.17	0.49	0.18	1.36
PROM (premature rupture of membranes) (1)	0.22	0.37	0.35	1.00	0.55	1.24	0.61	2.54
Chorioamnionitis (1)	−0.31	0.40	0.62	1.00	0.43	0.73	0.34	1.59
Multiple gestation (1)	22.85	40,192.97	0.00	1.00	1.00			
Constant	−0.91	0.23	16.29	1.00	0.00	0.40		

a. Variable(s) entered on step 1: Marital Status, Race/Ethnicity, Language Barrier (English not primary language), Type of Insurance, Prenatal Care, Anxiety/Depression, IDM, Hypertension, Obesity, Genital Tract Infection, PROM (premature rupture of membranes), Chorioamnionitis, Multiple Gestation.

### Logistic regression

The logistic regression analysis (Table 5) examining factors associated with birth trauma, compared to no trauma, included maternal sociodemographic, obstetric, and medical variables. After adjusting for all predictors, significant associations were observed for race/ethnicity, anxiety/depression, and hypertensive disorders. Mothers identifying as Race/Ethnicity group 4 had over five times the odds of experiencing birth trauma compared to the reference group (OR = 5.33, 95% CI: 1.08–26.27, *p* = 0.04). A history of anxiety or depression was associated with a 36% reduction in the odds of birth trauma (OR = 0.64, 95% CI: 0.43–0.96, *p* = 0.03). Conversely, chronic hypertension, gestational hypertension, or preeclampsia increased the odds of birth trauma by 70% (OR = 1.70, 95% CI: 1.12–2.56, *p* = 0.01). All other variables, including marital status, language barrier, type of insurance, prenatal care, obesity, genital tract infection, PROM, chorioamnionitis, and multiple gestation, were not statistically significant predictors in the model.

## Discussion

The incidence of major birth trauma, such as non-scalp injuries, has decreased over the years (2, 18). This study showed that scalp injuries were the most common form of neonatal birth trauma. Although previously considered benign, scalp injuries have been associated with increased morbidity, including longer hospital stays and, consequently, higher hospital charges (2). In our study, neonates who experienced birth trauma were less likely to require transfer to a higher-level care facility (23% vs. 39.3%, *p* < 0.001). Scalp injuries, being the most common in our study and generally benign in nature, may explain this finding.

Our study found that mothers who delivered neonates with birth trauma were younger, had fewer pregnancies, and had lower parity. Younger maternal age has been associated with skeletal immaturity and tighter pelvic musculature, which may contribute to birth trauma in some populations (7, 19, 20). However, in our cohort, the mean age (28.9 vs. 27.8 years, *p* = 0.042) and height (164.2 vs. 163.2 cm, *p* = 0.09) differences between groups fall within the range of full skeletal maturity and above thresholds typically linked to cephalopelvic disproportion. Thus, while the proposed mechanism is biologically plausible, these findings suggest it may not be clinically relevant in this sample and could represent a chance association.

Our initial hypothesis was that women with limited access to resources or care—such as those of lower socioeconomic status, those facing language barriers, or those without adequate prenatal care—would be at higher risk of delivering infants with birth trauma. We analyzed variables including race, presence of language barriers, type of insurance, marital status, access to prenatal care, and mean household income based on zip code. However, no statistically significant differences were observed between the groups. The six SDOH factors we selected may not fully capture the complexity of healthcare disparities, which could explain the lack of observed differences. A prospective study would allow for more comprehensive data collection, including variables not typically documented in medical records. Incorporating tools such as the Social Vulnerability Index, as used in a Canadian study (21), may provide deeper insight into the relationship between social factors and birth trauma. The hospital network involved in this study comprises nine locations in Central Illinois and one in Michigan, encompassing rural, suburban, and urban centers. Of these, eight facilities are equipped to provide Level II newborn nursery care, one offers only Level I care, and the primary hospital within the network can provide Level IV NICU services. For ease of data collection, our study included only neonates with unexpected complications. Expanding future research to include healthy neonates—thereby increasing the sample size—may offer a completer and more representative picture of potential disparities. Since the time of data collection, several smaller facilities in the region have closed, resulting in the emergence of obstetric care deserts. We emphasize the critical importance of maintaining access to local obstetric services to ensure optimal outcomes for both mothers and their infants.

In the U.S., up to 10% of pregnancies are affected by maternal hypertension (22, 23). These women are at increased risk for obstetric interventions such as earlier induction of labor or undergoing cesarean section. Since the neonates are physiologically less ready for birth, they have a higher risk of morbidities such as preterm birth, low birth weight, respiratory distress syndrome, and sepsis, leading to an increased need for NICU admission (24). Our study found that chronic or gestational hypertension, with or without pre-eclampsia, was more prevalent among mothers who delivered neonates with birth trauma compared to those without. To our knowledge, no previous studies have demonstrated an association between maternal hypertension and neonatal birth trauma.

About 20% of deliveries in the U.S. involve the use of FSE. It is an important tool for intrapartum fetal surveillance, particularly in cases of non-reassuring fetal heart rate tracings or when external monitoring is difficult due to maternal body habits or excessive fetal movement (25). However, FSE use increases the risk of scalp injury, cephalohematoma, and neonatal sepsis, regardless of the mode of delivery (1, 25). Our study also found that mothers with a history of FSE use had twice the odds of delivering neonates with birth trauma.

Shoulder dystocia, an obstetric emergency, occurs in up to 1% of spontaneous vaginal deliveries and 9% of operative vaginal deliveries (26, 27). It has been associated with brachial plexus injuries, fractures, and adverse neonatal outcomes such as HIE and death (1, 28). Our study similarly found that neonates who experienced shoulder dystocia had ten times higher odds of sustaining birth trauma.

The association between epidural analgesia and birth trauma observed in our study warrants further exploration. Among neonates who experienced birth trauma, 77.5% were born to mothers who received epidural anesthesia, compared to 51.7% in the non-trauma group. This disparity may be attributed to factors such as prolonged second stage of labor, abnormal fetal head position, and increased frequency of operative vaginal deliveries or cesarean sections (29–31). Although our dataset did not include detailed labor duration metrics, existing literature suggests that epidural analgesia is associated with longer second stage labor and a higher incidence of instrumental delivery—both of which are established risk factors for neonatal trauma.

Despite these associations, the overall risk to neonates exposed to maternal epidural anesthesia remains low. However, short-term effects such as lower Apgar scores at 1 and 5 min, increased need for resuscitation, higher NICU admission rates, and delayed initiation of breastfeeding have been reported (29–32). These results underscore the importance of careful intrapartum management and informed decision-making regarding analgesia use during labor.

Operative vaginal deliveries, such as those involving forceps or vacuum extraction, have been major risk factors for birth trauma. Common injuries—including scalp edema, bruising, cephalohematoma, subgaleal hemorrhage, and intracranial hemorrhage—are more prevalent with unsuccessful extractions (33, 34). Our study found that neonates born via operative vaginal delivery or emergent cesarean section had significantly higher odds of experiencing birth trauma. However, when vacuum and forceps deliveries were analyzed separately, we did not find a significant association between failed extraction and birth trauma.

Non-scalp injuries have been associated with higher odds of transient tachypnea of the newborn, meconium aspiration, respiratory distress syndrome, the need for CPAP or mechanical ventilation, HIE, seizures, and sepsis. Scalp injuries have additionally been shown to increase the odds of hyperbilirubinemia (2, 35). Our study found that neonates with birth trauma were less likely to require respiratory support, such as continuous positive airway pressure or non-invasive mechanical ventilation. Scalp injuries, which were the most common type of birth trauma in our study group, may explain the lower likelihood of requiring respiratory support. In contrast, the control group included neonates with unexpected complications, which may account for the observed association. Also, those with birth trauma were over four times more likely to have culture-proven sepsis or meningitis compared to those who did not experience trauma.

Meconium-stained amniotic fluid is a condition that requires notification and the presence of a Neonatal Advanced Life Support (NALS)-credentialed provider at delivery (36). In our study, the odds of experiencing birth trauma were reduced by more than half in the presence of MAS compared to those without MAS (5.9% vs. 11.8%, *p* = 0.021). This may be attributed to the advanced resuscitation skills of the provider attending. Additionally, neonates with birth trauma were significantly less likely to develop a pneumothorax compared to those without trauma (5.4% vs. 13.3%, *p* = 0.003). We hypothesize that this, too, may be due to the involvement of highly skilled providers, who are more likely to be present at high-risk deliveries such as cesarean sections and operative vaginal deliveries among others.

## Strength and limitations of this study

This study was conducted across 10 locations representing a mix of rural, suburban, and urban centers. These sites served populations with diverse socioeconomic backgrounds and offered varying levels of neonatal care. A key strength of the study lies in the diversity of settings and patient populations, which enhances the generalizability of the findings within the region.

However, several limitations should be acknowledged. First, the study was limited to the Midwest region, which may affect the applicability of the results to other geographic areas. As a retrospective study, data collection relied on the accuracy and completeness of existing medical records, which introduces potential variability. Additionally, the providers attending deliveries varied across sites and included general pediatricians, neonatal nurse practitioners, and neonatal fellows. In some locations, initial resuscitation efforts were initiated by obstetric staff before more advanced neonatal providers arrived, potentially contributing to inconsistencies in care across the network.

Selection bias is another important limitation. Rather than include all neonates born during the study period, we focused only on those with unexpected complications requiring admission to the Special Care Nursery or NICU. This enriched our study population with higher-risk neonates, which may make the observed rate of birth trauma appear higher than in a general newborn population. Moreover, this approach may have affected the distribution of social determinants of health (SDOH), as differences between complicated neonates with and without trauma are likely smaller than those between healthy neonates and those who experience trauma. While this decision was made in part to reduce the chart review burden, it introduces a potential source of selection bias that should be considered when interpreting the results.

## Conclusion

This study highlights important associations between maternal and labor/delivery factors and neonatal birth trauma. Factors such as young maternal age, low gravidity and parity, use of FSE, shoulder dystocia, and operative vaginal deliveries have been documented in the literature. However, SDOH did not appear to influence the incidence of neonatal birth trauma. The associations with maternal hypertension and epidural anesthesia warrant further investigation.

These findings can benefit both obstetricians and pediatricians/neonatologists in case management and prognostication, allowing for more informed decision-making and tailored care strategies.

## Data Availability

The original contributions presented in the study are included in the article/Supplementary Material, further inquiries can be directed to the corresponding author.
