# Supplementary material for: Neonatal birth trauma: identifying new risk factors and short-term outcomes
Source: Front Pediatr. 2025 Oct 7;13:1648252. doi: 10.3389/fped.2025.1648252 (PMC12537782; doi:10.3389/fped.2025.1648252)
Supplement: Supplementary file 1 [file Datasheet1.docx]

**Supplementary Materials**

Sup. table 1

| ***Birth Trauma Specific*** | ***N*** | ***Percentage*** |
| --- | --- | --- |
| Scalp injuries | 118 | 46% |
| Cerebral Hemorrhage | 9 | 4% |
| Skull fracture | 2 | 1% |
| Soft tissue injuries | 67 | 26% |
| Cranial Nerve injury | 5 | 2% |
| Fracture of clavicle | 14 | 5% |
| Fracture of long bones | 6 | 2% |
| Brachial plexus injury | 29 | 11% |
| Others | 6 | 2% |
| **All cases of Birth Trauma** | **256** | **100%** |
| **Individuals with at least One birth trauma** | **187** |  |
| ***Scalp specific*** | | |
| Cephalohematoma | 28 | 23% |
| Caput Succedaneum | 78 | 63% |
| Hemorrhage | 12 | 10% |
| Lesions of scalp | 5 | 4% |
| **All cases of Scalp Injuries** | **123** | **100%** |
| **Individuals with at least One scalp injury** | **113** |  |
| ***Cerebral Hemorrhage Specific*** | | |
| Subdural hemorrhage | 7 | 64% |
| Cerebral hemorrhage | 0 | 0% |
| Subarachnoid hemorrhage | 0 | 0% |
| Tentorial hemorrhage | 0 | 0% |
| Lacerations | 0 | 0% |
| Intracranial hemorrhage | 0 | 0% |
| Intraventricular hemorrhage | 4 | 36% |
| **All cases of Cerebral Hemorrhage** | **11** | **100%** |
| **Individuals with at least One Cerebral hemorrhage** | **9** |  |
| ***Soft tissue injuries specific*** | | |
| Erythema and abrasions | 18 | 23% |
| Petechiae | 7 | 9% |
| Ecchymoses and bruising | 51 | 64% |
| Subcutaneous fat necrosis | 1 | 1% |
| Lacerations | 3 | 4% |
| **All cases of soft tissue injuries** | **80** | **100%** |
| **Individuals with at least One soft tissues injury** | **67** |  |
